# CA19-9-producing esophageal adenocarcinoma originating from the esophageal cardia of the mid-thoracic esophagus: a case report

**DOI:** 10.1186/s40792-021-01252-1

**Published:** 2021-07-15

**Authors:** Naoki Kuwayama, Isamu Hoshino, Hisashi Gunji, Takeshi Kurosaki, Toru Tonooka, Hiroaki Soda, Itaru Sonoda, Ryotaro Eto, Nobuhiro Takiguchi, Yoshihiro Nabeya, Makiko Itami, Wataru Takayama

**Affiliations:** 1 Division of Gastroenterological Surgery, Chiba Cancer Center, 666-2 Nitona-cho, Chuo-ku, Chiba, 260-8717 Japan; 2 Division of Surgical Pathology, Chiba Cancer Center, 666-2 Nitona-cho, Chuo-ku, Chiba, 260-8717 Japan

**Keywords:** Esophageal adenocarcinoma, Esophageal cardiac glands, CA19-9

## Abstract

**Background:**

Although there are many studies on primary esophageal adenocarcinoma arising from Barrett's esophagus or ectopic gastric mucosa, reports on adenocarcinoma arising from esophageal cardiac glands are extremely rare. Herein, we report a case of mid-thoracic cancer antigen 19-9 (CA 19-9)-producing primary esophageal adenocarcinoma, which presumably originated from the cardiac glands.

**Case presentation:**

A 74-year-old man was referred to our department with advanced esophageal cancer, which initially presented with dyspepsia. Serum levels of cancer antigen 19-9 (CA 19-9) were elevated (724.89 U/ml). Upper gastrointestinal endoscopy revealed a type 2 tumor on the posterior wall of the mid-thoracic esophagus approximately 29–32 cm from the incisor. Mucosal biopsy was consistent with a diagnosis of adenocarcinoma. Contrast-enhanced computed tomography showed a circumferential wall thickening in the mid-thoracic esophagus without enlarged lymph nodes or distant metastasis. Positron emission tomography–computed tomography showed accumulation in the primary tumor, but no evidence of lymph node or distant metastasis. According to these findings, the adenocarcinoma was staged as cT3N0M0, thereby, requiring subtotal esophagectomy with lymph node dissection. Postoperative course was uneventful. Histopathologic analysis revealed a 50 × 40 mm moderately differentiated adenocarcinoma with invasion to the thoracic duct and lymph node metastasis at #108(1/4), #109R(1/3), and #109L(1/3). After surgery, the stage was revised to moderately differentiated pT4apN2pM0 (pStage III). Immunostaining revealed expression of CA19-9 and suggested esophageal cardiac gland origin of the tumor. Three months after the surgery, the patient showed no recurrence and is undergoing outpatient observation.

**Conclusions:**

We experienced a case of mid-thoracic CA19-9-producing primary esophageal adenocarcinoma, which was presumed to have originated in the esophageal cardiac glands. Due to the scarcity of studies regarding this condition, specific management needs to be further clarified.

## Background

Although the incidence of esophageal adenocarcinoma has increased in the recent years with the rise of Barrett's esophageal adenocarcinoma, primary esophageal adenocarcinoma arising in the mid-thoracic esophagus, that is those not derived from Barrett's esophagus or ectopic gastric mucosa, is extremely rare [1]. To the best of our knowledge, there has been no report of esophageal adenocarcinoma derived from esophageal cardiac glands with high CA19-9 levels. Herein, we describe a rare case of CA19-9-producing mid-thoracic esophageal adenocarcinoma, which likely originated from esophageal cardiac glands.

## Case presentation

**Fig. 1:**
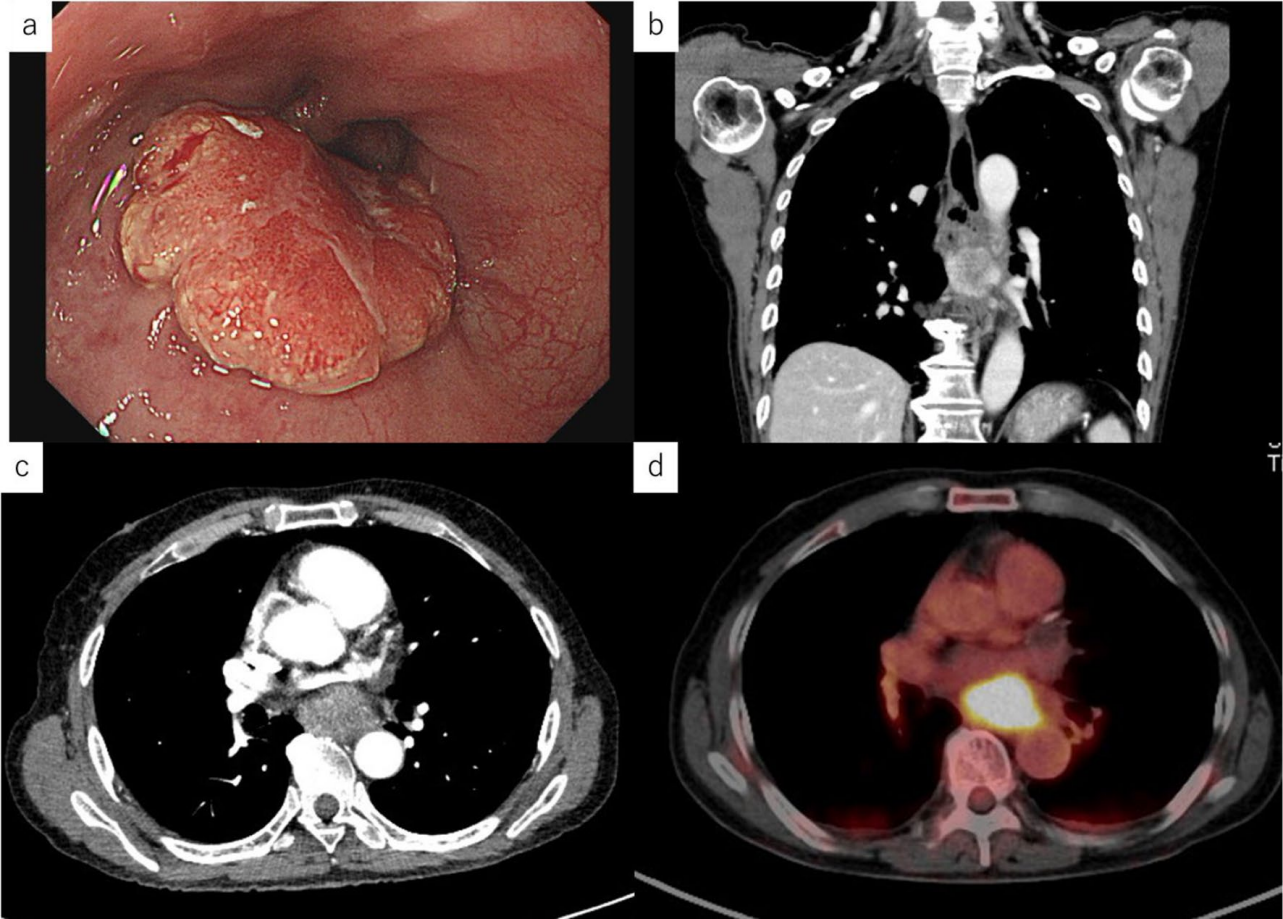
Test results of the patient. **a** Upper gastrointestinal endoscopy showing a type 2 tumor on the posterior wall of the mid-thoracic esophagus, approximately 29–32 cm from the incisor. **b**, **c** Contrast-enhanced computed tomography revealing a circumferential wall thickening in the mid-thoracic esophagus without enlarged lymph nodes or distant metastasis. **d** Positron emission tomography–computed tomography showing accumulation in the primary tumor, but no evidence of lymph node metastasis or distant metastasis

**Fig. 2:**
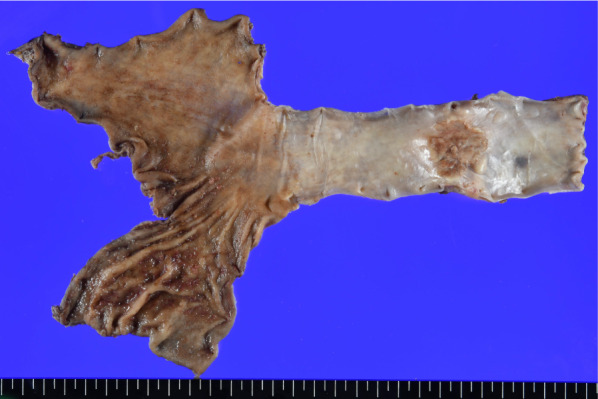
Histopathologic analysis revealed a 50 × 40 mm moderately differentiated adenocarcinoma with invasion to the thoracic duct and lymph node metastasis at #108(1/4), #109R(1/3), and #109L(1/3). After surgery, the stage was revised to moderately differentiated pT4apN2pM0 (pStage III)

**Fig. 3:**
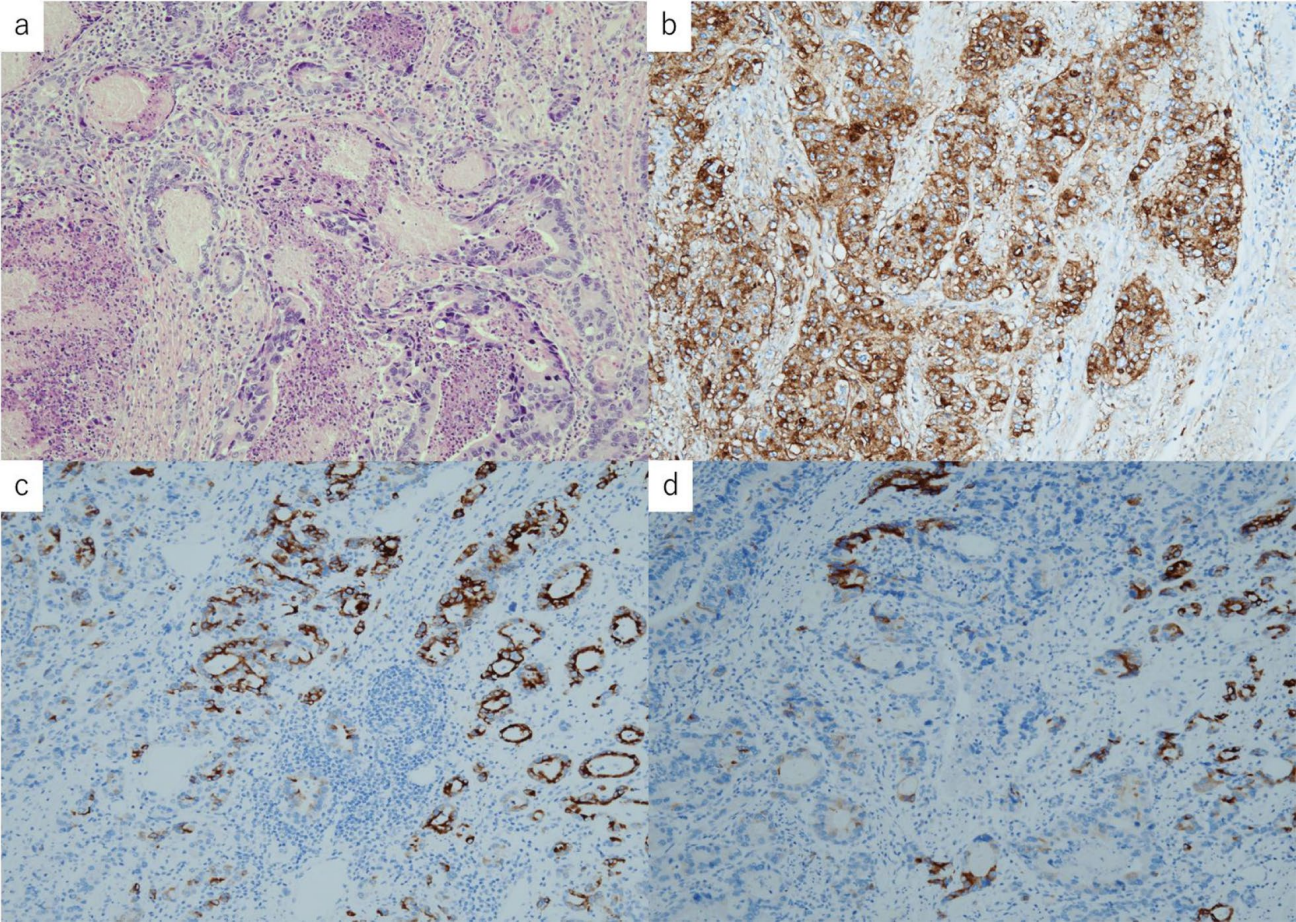
Histopathologic and immunostaining analysis. **a** Histopathologic analysis revealed a 50 × 40 mm moderately differentiated adenocarcinoma. **b** Immunostained sections showing increased diffuse expression of CA19-9 in the main tumor and in all three metastatic lymph nodes. **c**, **d** Immunostaining was also positive for MUC5AC (**c**), epithelial-type mucus core proteins of the gastric gland fossa, and MUC6 (**d**), a glandular-type mucus core protein present in the pyloric and cardiac glands

A 74-year-old man was referred to our department for advanced esophageal cancer, which initially presented with dyspepsia. Serum CA19-9 levels were elevated (724.89 U/ml). Upper gastrointestinal endoscopy revealed a type 2 tumor on the posterior wall of the mid-thoracic esophagus, approximately 29–32 cm from the incisor (Fig. 1a). Histopathologic analysis of the mucosal biopsy revealed adenocarcinoma. Contrast-enhanced computed tomography showed circumferential wall thickening in the mid-thoracic esophagus without enlarged lymph nodes and distant metastasis (Fig. 1b, c). Positron emission tomography–computed tomography showed accumulation in the primary tumor, but no evidence of lymph node or distant metastasis (Fig. 1d). Accordingly, a clinical stage of cT3N0M0 was established, thereby, requiring subtotal esophagectomy with lymph node dissection. The postoperative course was uneventful. Histopathologic analysis revealed a 50 × 40 mm moderately differentiated adenocarcinoma with invasion in the thoracic duct (pT4a) and lymph node metastasis at #108(1/4), #109R(1/3), and #109L(1/3) (pN2). These postoperative findings warranted the revision of the diagnosis and stage to moderately differentiated adenocarcinoma pT4apN2pM0 (pStage III) (Figs. 2 and 3a).

Immunostaining revealed the increased diffuse expression of CA19-9 in the main tumor and all three metastatic lymph nodes, suggesting that it was a CA19-9-producing tumor. Moreover, it was also positive for (1) MUC5AC, epithelial-type mucus core proteins of the gastric gland fossa and (2) MUC6, a glandular-type mucus core protein present in the pyloric and cardiac glands (Fig. 3c, d). Furthermore, histopathologically, no ectopic gastric mucosa was found in the background mucosa. The absence of Barrett's epithelium and ectopic gastric mucosa in the mid-thoracic region led us to speculate that the tumor originated from the esophageal cardia. Three months postoperatively, the patient showed no recurrence and is undergoing outpatient observation.

## Discussion

Primary esophageal adenocarcinoma is thought to originate from four sites: (1) esophageal glands proper, (2) esophageal cardiac glands, (3) ectopic gastric mucosa, and (4) Barrett's esophagus [1, 2]. The number of reports on esophageal adenocarcinoma has increased in the recent years, most of which are arising from Barrett's esophagus. Mid-thoracic primary esophageal adenocarcinoma, which is not arising from Barrett's esophagus or ectopic gastric mucosa, is an extremely rare disease. To the best of our knowledge, there has been no report of primary esophageal adenocarcinoma originating from the esophageal cardiac glands in the mid-thoracic region.

Esophageal cardiac glands are mucous glands found in the intrinsic layer of the esophageal mucosa, usually covered by superficial squamous epithelium [3]. The average width of these gland based on histopathologic examinations is around 4 mm (1–26 mm). This is continuously distributed from the gastric to the esophageal side. However, in cases of gastroesophageal reflux disease or short-segment Barrett’s esophagus, the glands are more extensive, reaching up to the squamous surface and are sometimes discontinuous [4, 5]. As a morphologic feature of the tumor, the esophageal cardiac glands are located in the intrinsic layer of the mucosa. In contrast, esophageal glands originate from the submucosal tissue. It has been reported that tumors derived from esophageal glands tend to be more deeply invasive such that these often develop in a submucosal tumor-like fashion [6]. In the present case, it was difficult to infer the origin of the tumor based on the morphologic characteristics of the tumor alone. However, immunohistochemical analysis had aided in the establishment of its etiology. Results revealed that the primary tumor had increased diffuse expression of MUC5AC and MUC6. MUC5AC is gastric epithelial-type mucus core protein found in the surface epithelium of normal gastric mucosa. In contrast, MUC6 is a pyloric gland-type mucus core protein found in pyloric and cardiac glands. These MUCs are usually absent in esophageal intrinsic glands [7, 8]. Moreover, the absence of Barrett's epithelium and the rare occurrence of ectopic gastric mucosa in the mid-thoracic region led us to speculate that the tumor originated from the esophageal cardia [9].

In addition, due to the increased preoperative serum CA19-9 levels and the positive CA19-9 expression in the tumor and metastatic lymph nodes during immunostaining, this tumor was suspected to be a CA19-9-producing tumor. Although CA19-9 is a widely used tumor marker for gastrointestinal cancers (for example, gastric, colorectal, pancreatic, and biliary cancers), its exact significance in esophageal adenocarcinoma has not been clarified [10]. Tokunaga et al. found that for adenocarcinoma of the esophagogastric junction, carcinoembryonic antigen (CEA) and CA19-9 positivity rates were significantly higher (*P* = 0.002 and < 0.001, respectively) in patients with myometrial and deeper tumor invasion (*P* ≤ 0.0063 [= 0.05/8]). Moreover, CA19-9 was associated with higher cancer-specific survival (multivariate hazard ratio [HR] = 3.89, 95% confidence interval [CI] 1.41–10.33; *P* = 0.010) and overall survival (multivariate HR = 2.43, 95% CI 1.03–5.35; *P* = 0.043) as independent prognostic factors [11]. Scarpa et al. reported that serum levels of CA19-9 and CEA were significantly higher in patients with unresectable esophageal adenocarcinoma compared to those with resectable esophageal adenocarcinoma (*P* = 0.001 and *P* = 0.003, respectively), indicating that these were significant biomarkers of highly advanced cancer [12]. Van der Kaaij et al. found that median overall survival was different among patients with normal levels of both CEA and CA19-9 (*n* = 59, 51 months), elevated levels of CA19-9 only (*n* = 19, 24 months), and elevated levels of both CEA and CA19-9 (*n* = 11, 11 months) (*P* < 0.001). They reported that the median time to tumor recurrence was 34 months in patients with normal CEA and CA19-9 levels and 7 months in patients with elevated levels (*P* = 0.003) [13].

Although further prospective validation is needed, CA19-9 is a useful marker for estimating the progression and prognosis of patients with esophageal adenocarcinoma. Together with other clinical information, it has the potential for identifying patients who will likely benefit from early systemic treatment. Regarding the choice of treatment for the patient, we chose upfront surgery for a preoperative diagnosis of cT3N0M0. This was considered because of the patient's relatively old age, difficulty in oral intake, and significant weight loss, which made it difficult to complete the preoperative treatment. In general, the efficacy of preoperative chemotherapy and chemoradiation has been reported for both squamous cell carcinoma and adenocarcinoma in locally advanced esophageal cancer without distant metastasis and those with nodal metastasis [14, 15].

Recently, CA19-9 has been found to be specifically elevated in pancreatic cancer recurrence and is expected to be useful for surveillance of adenocarcinoma [16].

In this case, CA19-9 levels remained high (647.49 U/ml) after surgery. It is reported that CA19-9 is an independent prognostic factor in gastric cancer [17], and that patients whose CA19-9 does not normalize after resection have a poor prognosis [18]. There are no reports on the usefulness of CA19-9 for postoperative surveillance of esophageal adenocarcinoma, and further accumulation of cases is needed. Thus, careful follow-up is necessary in this case as well.

## Conclusions

In conclusion, most of the previous reports have focused on esophageal adenocarcinoma derived from Barrett's esophagus in various locations (lower thoracic, abdominal, and junctional). We experienced a case of CA19-9-producing primary esophageal adenocarcinoma, which was presumed to have originated from the esophageal cardiac glands of the mid-thoracic esophagus. Due to the scarcity of studies regarding CA19-9-producing adenocarcinoma derived from the cardiac glands in the mid-thoracic esophagus, the specific treatment is still unknown. Further studies are crucial for establishing safe and effective approaches for adequate management.

## Data Availability

Data sharing is not applicable to this article, as no data sets were generated or analyzed during the study.

## Abbreviations

CA 19-9: Cancer antigen 19-9
CEA: Carcinoembryonic antigen
HR: Hazard ratio
CI: Confidence interval
